# 1-Bromo-2,4,6-tricyclo­hexyl­benzene

**DOI:** 10.1107/S1600536807067062

**Published:** 2007-12-21

**Authors:** Joel T. Mague, Lisa Linhardt, Iliana Medina, Mark J. Fink

**Affiliations:** a Department of Chemistry, Tulane University, New Orleans, LA 70118, USA

## Abstract

The title compound, C_24_H_25_Br, packs efficiently in the crystal structure with no solvent-accessible voids and several inter­molecular H⋯H contacts approximating the sum of the van der Waals radii. The mol­ecule is quite crowded, with intra­molecular Br⋯H and C⋯H contacts *ca* 0.38 and 0.30 Å, respectively, less than the sum of the corresponding van der Waals radii. All cyclo­hexyl rings adopt chair conformations with the ‘seat’ of the chair inclined at approximately 57–81° to the mean plane of the benzene ring, while those *ortho* to bromine have their centroids displaced in opposite directions from this plane.

## Related literature

For related structures see: Columbus *et al.* (1994 ▶); Vilardo *et al.* (2000 ▶). For the synthesis see: Kouldelka *et al.* (1985 ▶). For related literature, see: Saito *et al.* (2004 ▶).
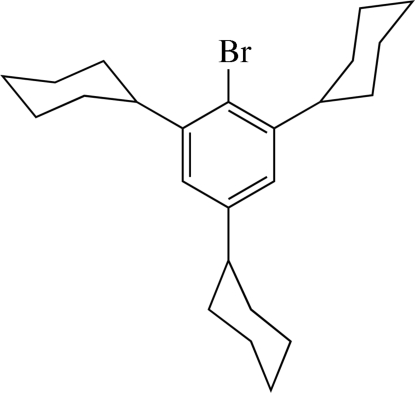


## Experimental

### 

#### Crystal data

C_24_H_35_Br
*M* *_r_* = 403.43
Monoclinic, 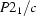

*a* = 15.510 (1) Å
*b* = 11.6718 (8) Å
*c* = 11.3431 (8) Å
β = 99.912 (1)°
*V* = 2022.7 (2) Å^3^
*Z* = 4
Mo *K*α radiation
μ = 2.04 mm^−1^
*T* = 100 (2) K
0.20 × 0.11 × 0.04 mm

#### Data collection

Bruker SMART APEX CCD area-detector diffractometer
Absorption correction: multi-scan (*SADABS*; Sheldrick, 2002 ▶) *T*_min_ = 0.742, *T*_max_ = 0.921
17176 measured reflections
4621 independent reflections
3711 reflections with *I* > 2σ(*I*)
*R*_int_ = 0.044

#### Refinement

*R*[*F* ^2^ > 2σ(*F* ^2^)] = 0.033
*wR*(*F* ^2^) = 0.090
*S* = 1.08
4621 reflections
226 parameters
H-atom parameters constrained
Δρ_max_ = 0.48 e Å^−3^
Δρ_min_ = −0.32 e Å^−3^

### 

Data collection: *SMART* (Bruker, 2000 ▶); cell refinement: *SAINT-Plus* (Bruker, 2004 ▶); data reduction: *SAINT-Plus*; program(s) used to solve structure: *SHELXS97* (Sheldrick, 1997 ▶); program(s) used to refine structure: *SHELXL97* (Sheldrick, 1997 ▶); molecular graphics: *SHELXTL* (Bruker, 2000 ▶); software used to prepare material for publication: *SHELXTL*.

## Supplementary Material

Crystal structure: contains datablocks I, global. DOI: 10.1107/S1600536807067062/lh2584sup1.cif

Structure factors: contains datablocks I. DOI: 10.1107/S1600536807067062/lh2584Isup2.hkl

Additional supplementary materials:  crystallographic information; 3D view; checkCIF report

## Figures and Tables

**Table 1:** Cremer & Pople (1975 ▶) puckering parameters (Å,°)

Ring	*Q*	θ	ϕ
C7–C12	0.586	179.6	279.2
C13–C18	0.574	2.5	345.0
C19–C24	0.577	178.2	220.7

**Fig. 1.**
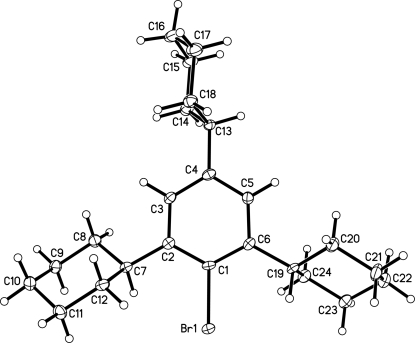
Molecular structure of I. Displacement ellipsoids are drawn at the 50% probability level. H-atoms are drawn as spheres of arbitrary radius.

## supplementary crystallographic information

### Comment

The title compound (TCBBr, I) has been prepared as a precursor to the Grignard reagent TCBMgBr (Kouldelka *et al.*, 1985) with the latter used to synthesize sterically hindered acetophenones and nitrobenzenes as well as stable stannylenes (*R*_2_Sn:), stannanethiones (*R*_2_Sn=S) and stannaneselenones (*R*_2_Sn=Se) (Saito, *et al.*, 2004). Viewed along the Br-C1vector, the plane defined by C8, C9, C11 and C12 ("seat" of the chair) is inclined at an angle of 57.3 (3)° to the plane of the aromatic ring while C7 is 0.07 (1) Å below the latter plane. Similarly, the plane defined by C20, C21, C23 and C24 is inclined at an angle of 122.4 (3)° with C19 0.07 (1) Å below the plane of the aromatic ring while the plane defined by C14, C15, C17, C18 makes an angle of 81.5 (3)° with the latter plane. The tilt is towards C5 and C13 lies in the plane of the aromatic ring. Additionally, the center of gravity of the C7—C12 ring lies 0.23 (1) Å above the mean plane of the aromatic ring while that of the C19—C24 ring lies 0.51 (1) Å below it. This contrasts with 2,6-dicyclohexyl-3,5-di-*tert*-butylphenol (Vilardo *et al.*, 2000) and 2,3,6-tricyclohexylbiphenyl (Columbus *et al.*, 1994) where the centers of gravity of the corresponding cyclohexyl groups are essentially in the plane of the aromatic ring (in the former this is required by symmetry). The methine H atoms H7 and H19 point towards the bromine which is the orientation seen in 2,3,6-tricyclohexylbiphenyl but opposite from that in 2,6-dicyclohexyl-3,5-di-*tert*-butylphenol. All three cyclohexyl groups adopt chair conformations with the pertinent puckering parameters (Cremer & Pople, 1975) listed in Table 1. There are 20 intermolecular H···H contacts that are 0.09 (2)–0.13 (2) Å less than the sum of the van der Waals radii and 14 equal to this sum indicative of the compact molecular packing. In addition there are 12 intramolecular H···C contacts 0.07 (2)–0.30 (2) Å less and 9 intramolecular H···H contacts 0.05 (3)–0.30 (3) Å less than the sums of the respective van der Waals radii. This contrasts with 2,6-dicyclohexyl-3,5-di-*tert* -butylphenol where no such short contacts are seen and 2,3,6-tricyclohexylbiphenyl where there is one C—H···*Cg* (*Cg* is the center of gravity of the central aromatic ring) interaction with H···*Cg* = 2.71 Å and a C—H···*Cg* angle of 161°.

### Experimental

The title compound was prepared by the literature method (Kouldelka *et al.*, 1985).

### Refinement

H atoms were included in calculated postions with C—H = 0.95 - 1.00Å and were included in the riding-model approximation with *U*_iso_(H) = 1.2*U*_eq_(C).

### Crystal data

**Table:**

C_24_H_35_Br	*F*_000_ = 856
*M**_r_* = 403.43	*D*_x_ = 1.325 Mg m^−^^3^
Monoclinic, *P*2_1_/*c*	Mo *K*α radiation λ = 0.71073 Å
Hall symbol: -P 2ybc	Cell parameters from 7741 reflections
*a* = 15.510 (1) Å	θ = 2.2–28.2º
*b* = 11.6718 (8) Å	µ = 2.04 mm^−^^1^
*c* = 11.3431 (8) Å	*T* = 100 (2) K
β = 99.912 (1)º	Plate, colorless
*V* = 2022.7 (2) Å^3^	0.20 × 0.11 × 0.04 mm
*Z* = 4	

### Data collection

**Table:**

Bruker SMART APEXI CCD area-detector diffractometer	4621 independent reflections
Radiation source: fine-focus sealed tube	3711 reflections with *I* > 2σ(*I*)
Monochromator: graphite	*R*_int_ = 0.044
*T* = 100(2) K	θ_max_ = 27.5º
phi and ω scans	θ_min_ = 1.3º
Absorption correction: multi-scan(SADABS; Sheldrick, 2002)	*h* = −20→20
*T*_min_ = 0.742, *T*_max_ = 0.921	*k* = −15→15
17176 measured reflections	*l* = −14→14

### Refinement

**Table:**

Refinement on *F*^2^	Secondary atom site location: difference Fourier map
Least-squares matrix: full	Hydrogen site location: inferred from neighbouring sites
*R*[*F*^2^ > 2σ(*F*^2^)] = 0.033	H-atom parameters constrained
*wR*(*F*^2^) = 0.090	*w* = 1/[σ^2^(*F*_o_^2^) + (0.0478*P*)^2^] where *P* = (*F*_o_^2^ + 2*F*_c_^2^)/3
*S* = 1.08	(Δ/σ)_max_ = 0.001
4621 reflections	Δρ_max_ = 0.48 e Å^−^^3^
226 parameters	Δρ_min_ = −0.32 e Å^−^^3^
Primary atom site location: structure-invariant direct methods	Extinction correction: none

### Special details

**Table:**

Experimental. The diffraction data were collected in three sets of 606 frames (ω scans, 0.3°/scan) at φ settings of 0, 120 and 240°.
Geometry. All e.s.d.'s (except the e.s.d. in the dihedral angle between two l.s. planes) are estimated using the full covariance matrix. The cell e.s.d.'s are taken into account individually in the estimation of e.s.d.'s in distances, angles and torsion angles; correlations between e.s.d.'s in cell parameters are only used when they are defined by crystal symmetry. An approximate (isotropic) treatment of cell e.s.d.'s is used for estimating e.s.d.'s involving l.s. planes.
Refinement. Refinement of *F*^2^ against ALL reflections. The weighted *R*-factor *wR* and goodness of fit *S* are based on *F*^2^, conventional *R*-factors *R* are based on *F*, with *F* set to zero for negative *F*^2^. The threshold expression of *F*^2^ > σ(*F*^2^) is used only for calculating *R*-factors(gt) *etc*. and is not relevant to the choice of reflections for refinement. *R*-factors based on *F*^2^ are statistically about twice as large as those based on *F*, and *R*- factors based on ALL data will be even larger. H-atoms were placed in calculated positions (C—H = 0.95 - 0.98 Å) and included as riding contributions with isotropic displacement parameters 1.2 - 1.5 times those of the attached carbon atoms.

### Fractional atomic coordinates and isotropic or equivalent isotropic displacement parameters (Å^2^)

**Table:**

	*x*	*y*	*z*	*U*_iso_*/*U*_eq_	
Br1	0.629009 (15)	0.56395 (2)	1.082654 (19)	0.01661 (8)	
C1	0.68387 (15)	0.56480 (19)	0.94340 (19)	0.0126 (4)	
C2	0.63806 (15)	0.61497 (19)	0.8383 (2)	0.0131 (5)	
C3	0.68065 (15)	0.61665 (19)	0.7398 (2)	0.0138 (5)	
H3	0.6525	0.6525	0.6682	0.017*	
C4	0.76278 (15)	0.56804 (19)	0.7419 (2)	0.0138 (5)	
C5	0.80369 (15)	0.51719 (19)	0.8475 (2)	0.0135 (5)	
H5	0.8595	0.4830	0.8494	0.016*	
C6	0.76628 (15)	0.51420 (19)	0.95094 (19)	0.0126 (5)	
C7	0.54657 (15)	0.66413 (19)	0.83056 (19)	0.0134 (5)	
H7	0.5160	0.6171	0.8845	0.016*	
C8	0.49112 (15)	0.6580 (2)	0.7052 (2)	0.0176 (5)	
H8A	0.5194	0.7036	0.6489	0.021*	
H8B	0.4876	0.5775	0.6773	0.021*	
C9	0.39895 (15)	0.7037 (2)	0.7055 (2)	0.0178 (5)	
H9A	0.3649	0.7007	0.6233	0.021*	
H9B	0.3691	0.6547	0.7572	0.021*	
C10	0.40203 (16)	0.8265 (2)	0.7510 (2)	0.0192 (5)	
H10A	0.3418	0.8537	0.7531	0.023*	
H10B	0.4278	0.8767	0.6958	0.023*	
C11	0.45671 (15)	0.8340 (2)	0.8763 (2)	0.0183 (5)	
H11A	0.4281	0.7890	0.9328	0.022*	
H11B	0.4601	0.9147	0.9033	0.022*	
C12	0.54896 (15)	0.78791 (19)	0.8769 (2)	0.0154 (5)	
H12A	0.5825	0.7907	0.9594	0.019*	
H12B	0.5793	0.8371	0.8259	0.019*	
C13	0.80663 (15)	0.56872 (19)	0.63217 (19)	0.0129 (4)	
H13	0.8599	0.5193	0.6508	0.015*	
C14	0.74858 (16)	0.5171 (2)	0.5220 (2)	0.0178 (5)	
H14A	0.7309	0.4386	0.5409	0.021*	
H14B	0.6949	0.5639	0.5008	0.021*	
C15	0.79693 (17)	0.5128 (2)	0.4158 (2)	0.0207 (5)	
H15A	0.7569	0.4834	0.3447	0.025*	
H15B	0.8469	0.4591	0.4339	0.025*	
C16	0.83072 (18)	0.6307 (2)	0.3873 (2)	0.0245 (6)	
H16A	0.7805	0.6815	0.3583	0.029*	
H16B	0.8659	0.6233	0.3226	0.029*	
C17	0.88686 (17)	0.6843 (2)	0.4972 (2)	0.0215 (5)	
H17A	0.9037	0.7629	0.4776	0.026*	
H17B	0.9411	0.6389	0.5198	0.026*	
C18	0.83754 (16)	0.6886 (2)	0.6028 (2)	0.0167 (5)	
H18A	0.7863	0.7400	0.5830	0.020*	
H18B	0.8763	0.7203	0.6737	0.020*	
C19	0.81235 (15)	0.45326 (19)	1.06289 (19)	0.0137 (5)	
H19	0.7958	0.4936	1.1335	0.016*	
C20	0.91201 (15)	0.4548 (2)	1.0778 (2)	0.0204 (5)	
H20A	0.9327	0.5351	1.0789	0.024*	
H20B	0.9304	0.4158	1.0087	0.024*	
C21	0.95395 (16)	0.3951 (2)	1.1937 (2)	0.0230 (6)	
H21A	1.0184	0.3957	1.1999	0.028*	
H21B	0.9392	0.4376	1.2631	0.028*	
C22	0.92215 (16)	0.2718 (2)	1.1973 (2)	0.0231 (6)	
H22A	0.9473	0.2368	1.2751	0.028*	
H22B	0.9425	0.2270	1.1332	0.028*	
C23	0.82232 (16)	0.2673 (2)	1.1803 (2)	0.0190 (5)	
H23A	0.8030	0.1864	1.1768	0.023*	
H23B	0.8026	0.3038	1.2498	0.023*	
C24	0.78019 (16)	0.3286 (2)	1.0658 (2)	0.0171 (5)	
H24A	0.7158	0.3281	1.0603	0.021*	
H24B	0.7944	0.2867	0.9957	0.021*	

### Atomic displacement parameters (Å^2^)

**Table:**

	*U*^11^	*U*^22^	*U*^33^	*U*^12^	*U*^13^	*U*^23^
Br1	0.01767 (13)	0.02172 (14)	0.01130 (12)	0.00188 (10)	0.00488 (9)	0.00310 (10)
C1	0.0161 (11)	0.0139 (11)	0.0091 (10)	−0.0030 (9)	0.0054 (9)	0.0000 (9)
C2	0.0145 (11)	0.0122 (11)	0.0125 (11)	−0.0019 (9)	0.0016 (9)	−0.0002 (9)
C3	0.0172 (12)	0.0139 (11)	0.0098 (10)	0.0000 (9)	0.0008 (9)	0.0007 (9)
C4	0.0176 (11)	0.0126 (11)	0.0113 (10)	−0.0037 (9)	0.0027 (9)	0.0001 (9)
C5	0.0135 (11)	0.0132 (11)	0.0133 (11)	0.0011 (9)	0.0009 (9)	−0.0009 (9)
C6	0.0157 (11)	0.0106 (11)	0.0107 (11)	−0.0024 (9)	−0.0001 (9)	0.0007 (9)
C7	0.0155 (11)	0.0148 (12)	0.0101 (10)	−0.0004 (9)	0.0032 (9)	0.0011 (9)
C8	0.0185 (12)	0.0201 (12)	0.0131 (11)	0.0033 (10)	−0.0002 (9)	−0.0034 (10)
C9	0.0144 (12)	0.0232 (13)	0.0149 (11)	0.0014 (10)	−0.0004 (9)	0.0012 (10)
C10	0.0185 (13)	0.0186 (12)	0.0208 (12)	0.0043 (10)	0.0048 (10)	0.0041 (10)
C11	0.0213 (13)	0.0138 (12)	0.0205 (12)	0.0023 (10)	0.0055 (10)	−0.0009 (10)
C12	0.0194 (12)	0.0155 (12)	0.0119 (11)	−0.0008 (9)	0.0040 (9)	−0.0002 (9)
C13	0.0153 (11)	0.0141 (11)	0.0096 (10)	0.0016 (9)	0.0030 (9)	0.0002 (9)
C14	0.0210 (13)	0.0185 (12)	0.0137 (11)	0.0012 (10)	0.0024 (10)	−0.0004 (10)
C15	0.0266 (14)	0.0247 (14)	0.0110 (12)	0.0003 (11)	0.0042 (10)	−0.0021 (10)
C16	0.0351 (15)	0.0245 (14)	0.0162 (12)	0.0034 (12)	0.0110 (11)	0.0046 (11)
C17	0.0271 (14)	0.0179 (12)	0.0217 (13)	0.0002 (10)	0.0101 (11)	0.0033 (10)
C18	0.0211 (12)	0.0141 (11)	0.0157 (11)	0.0001 (10)	0.0054 (9)	0.0003 (9)
C19	0.0174 (12)	0.0168 (12)	0.0068 (10)	0.0000 (9)	0.0015 (9)	0.0008 (9)
C20	0.0147 (12)	0.0281 (14)	0.0175 (12)	−0.0021 (10)	0.0005 (10)	0.0077 (10)
C21	0.0165 (13)	0.0308 (14)	0.0192 (13)	−0.0010 (11)	−0.0040 (10)	0.0094 (11)
C22	0.0220 (13)	0.0277 (14)	0.0189 (13)	0.0060 (11)	0.0016 (10)	0.0072 (11)
C23	0.0226 (13)	0.0180 (12)	0.0160 (12)	0.0001 (10)	0.0022 (10)	0.0035 (10)
C24	0.0187 (12)	0.0171 (12)	0.0149 (11)	−0.0012 (10)	0.0011 (9)	0.0003 (9)

### Geometric parameters (Å, °)

**Table:**

Br1—C1	1.919 (2)	C14—C15	1.526 (3)
C1—C6	1.397 (3)	C14—H14A	0.9900
C1—C2	1.406 (3)	C14—H14B	0.9900
C2—C3	1.393 (3)	C15—C16	1.526 (4)
C2—C7	1.519 (3)	C15—H15A	0.9900
C3—C4	1.391 (3)	C15—H15B	0.9900
C3—H3	0.9500	C16—C17	1.526 (4)
C4—C5	1.388 (3)	C16—H16A	0.9900
C4—C13	1.518 (3)	C16—H16B	0.9900
C5—C6	1.396 (3)	C17—C18	1.529 (3)
C5—H5	0.9500	C17—H17A	0.9900
C6—C19	1.522 (3)	C17—H17B	0.9900
C7—C8	1.532 (3)	C18—H18A	0.9900
C7—C12	1.536 (3)	C18—H18B	0.9900
C7—H7	1.0000	C19—C20	1.526 (3)
C8—C9	1.526 (3)	C19—C24	1.541 (3)
C8—H8A	0.9900	C19—H19	1.0000
C8—H8B	0.9900	C20—C21	1.531 (3)
C9—C10	1.522 (3)	C20—H20A	0.9900
C9—H9A	0.9900	C20—H20B	0.9900
C9—H9B	0.9900	C21—C22	1.524 (4)
C10—C11	1.527 (3)	C21—H21A	0.9900
C10—H10A	0.9900	C21—H21B	0.9900
C10—H10B	0.9900	C22—C23	1.528 (3)
C11—C12	1.527 (3)	C22—H22A	0.9900
C11—H11A	0.9900	C22—H22B	0.9900
C11—H11B	0.9900	C23—C24	1.527 (3)
C12—H12A	0.9900	C23—H23A	0.9900
C12—H12B	0.9900	C23—H23B	0.9900
C13—C14	1.532 (3)	C24—H24A	0.9900
C13—C18	1.534 (3)	C24—H24B	0.9900
C13—H13	1.0000		
			
C6—C1—C2	123.5 (2)	C15—C14—H14B	109.5
C6—C1—Br1	118.56 (16)	C13—C14—H14B	109.5
C2—C1—Br1	117.97 (16)	H14A—C14—H14B	108.0
C3—C2—C1	116.5 (2)	C14—C15—C16	111.8 (2)
C3—C2—C7	121.0 (2)	C14—C15—H15A	109.3
C1—C2—C7	122.5 (2)	C16—C15—H15A	109.3
C4—C3—C2	122.7 (2)	C14—C15—H15B	109.3
C4—C3—H3	118.7	C16—C15—H15B	109.3
C2—C3—H3	118.7	H15A—C15—H15B	107.9
C5—C4—C3	118.1 (2)	C15—C16—C17	111.4 (2)
C5—C4—C13	120.5 (2)	C15—C16—H16A	109.3
C3—C4—C13	121.5 (2)	C17—C16—H16A	109.3
C4—C5—C6	122.8 (2)	C15—C16—H16B	109.3
C4—C5—H5	118.6	C17—C16—H16B	109.3
C6—C5—H5	118.6	H16A—C16—H16B	108.0
C5—C6—C1	116.5 (2)	C16—C17—C18	111.2 (2)
C5—C6—C19	120.6 (2)	C16—C17—H17A	109.4
C1—C6—C19	122.8 (2)	C18—C17—H17A	109.4
C2—C7—C8	113.94 (18)	C16—C17—H17B	109.4
C2—C7—C12	111.61 (18)	C18—C17—H17B	109.4
C8—C7—C12	109.78 (18)	H17A—C17—H17B	108.0
C2—C7—H7	107.0	C17—C18—C13	110.95 (19)
C8—C7—H7	107.0	C17—C18—H18A	109.5
C12—C7—H7	107.0	C13—C18—H18A	109.5
C9—C8—C7	110.99 (19)	C17—C18—H18B	109.5
C9—C8—H8A	109.4	C13—C18—H18B	109.5
C7—C8—H8A	109.4	H18A—C18—H18B	108.0
C9—C8—H8B	109.4	C6—C19—C20	114.07 (19)
C7—C8—H8B	109.4	C6—C19—C24	110.62 (18)
H8A—C8—H8B	108.0	C20—C19—C24	109.54 (19)
C10—C9—C8	110.8 (2)	C6—C19—H19	107.4
C10—C9—H9A	109.5	C20—C19—H19	107.4
C8—C9—H9A	109.5	C24—C19—H19	107.4
C10—C9—H9B	109.5	C19—C20—C21	111.2 (2)
C8—C9—H9B	109.5	C19—C20—H20A	109.4
H9A—C9—H9B	108.1	C21—C20—H20A	109.4
C9—C10—C11	110.45 (19)	C19—C20—H20B	109.4
C9—C10—H10A	109.6	C21—C20—H20B	109.4
C11—C10—H10A	109.6	H20A—C20—H20B	108.0
C9—C10—H10B	109.6	C22—C21—C20	111.2 (2)
C11—C10—H10B	109.6	C22—C21—H21A	109.4
H10A—C10—H10B	108.1	C20—C21—H21A	109.4
C10—C11—C12	110.75 (19)	C22—C21—H21B	109.4
C10—C11—H11A	109.5	C20—C21—H21B	109.4
C12—C11—H11A	109.5	H21A—C21—H21B	108.0
C10—C11—H11B	109.5	C21—C22—C23	110.7 (2)
C12—C11—H11B	109.5	C21—C22—H22A	109.5
H11A—C11—H11B	108.1	C23—C22—H22A	109.5
C11—C12—C7	111.23 (19)	C21—C22—H22B	109.5
C11—C12—H12A	109.4	C23—C22—H22B	109.5
C7—C12—H12A	109.4	H22A—C22—H22B	108.1
C11—C12—H12B	109.4	C24—C23—C22	111.6 (2)
C7—C12—H12B	109.4	C24—C23—H23A	109.3
H12A—C12—H12B	108.0	C22—C23—H23A	109.3
C4—C13—C14	112.55 (19)	C24—C23—H23B	109.3
C4—C13—C18	112.58 (18)	C22—C23—H23B	109.3
C14—C13—C18	110.07 (19)	H23A—C23—H23B	108.0
C4—C13—H13	107.1	C23—C24—C19	111.56 (19)
C14—C13—H13	107.1	C23—C24—H24A	109.3
C18—C13—H13	107.1	C19—C24—H24A	109.3
C15—C14—C13	110.94 (19)	C23—C24—H24B	109.3
C15—C14—H14A	109.5	C19—C24—H24B	109.3
C13—C14—H14A	109.5	H24A—C24—H24B	108.0
			
C6—C1—C2—C3	−2.6 (3)	C2—C7—C12—C11	−176.34 (18)
Br1—C1—C2—C3	178.47 (16)	C8—C7—C12—C11	56.3 (2)
C6—C1—C2—C7	176.8 (2)	C5—C4—C13—C14	125.1 (2)
Br1—C1—C2—C7	−2.1 (3)	C3—C4—C13—C14	−54.1 (3)
C1—C2—C3—C4	2.3 (3)	C5—C4—C13—C18	−109.8 (2)
C7—C2—C3—C4	−177.1 (2)	C3—C4—C13—C18	71.0 (3)
C2—C3—C4—C5	−0.7 (3)	C4—C13—C14—C15	−176.62 (19)
C2—C3—C4—C13	178.5 (2)	C18—C13—C14—C15	56.9 (3)
C3—C4—C5—C6	−0.8 (3)	C13—C14—C15—C16	−55.6 (3)
C13—C4—C5—C6	180.0 (2)	C14—C15—C16—C17	54.2 (3)
C4—C5—C6—C1	0.5 (3)	C15—C16—C17—C18	−54.5 (3)
C4—C5—C6—C19	177.6 (2)	C16—C17—C18—C13	56.5 (3)
C2—C1—C6—C5	1.2 (3)	C4—C13—C18—C17	176.06 (19)
Br1—C1—C6—C5	−179.83 (16)	C14—C13—C18—C17	−57.5 (3)
C2—C1—C6—C19	−175.8 (2)	C5—C6—C19—C20	30.8 (3)
Br1—C1—C6—C19	3.1 (3)	C1—C6—C19—C20	−152.3 (2)
C3—C2—C7—C8	28.9 (3)	C5—C6—C19—C24	−93.2 (3)
C1—C2—C7—C8	−150.4 (2)	C1—C6—C19—C24	83.7 (3)
C3—C2—C7—C12	−96.1 (2)	C6—C19—C20—C21	178.4 (2)
C1—C2—C7—C12	84.5 (3)	C24—C19—C20—C21	−57.0 (3)
C2—C7—C8—C9	177.37 (19)	C19—C20—C21—C22	57.6 (3)
C12—C7—C8—C9	−56.6 (3)	C20—C21—C22—C23	−55.6 (3)
C7—C8—C9—C10	57.7 (3)	C21—C22—C23—C24	54.8 (3)
C8—C9—C10—C11	−57.3 (3)	C22—C23—C24—C19	−55.6 (3)
C9—C10—C11—C12	56.9 (3)	C6—C19—C24—C23	−177.29 (19)
C10—C11—C12—C7	−56.8 (3)	C20—C19—C24—C23	56.1 (2)

### Table 1 Cremer &amp; Pople (1975) puckering parameters (Å,°)

**Table:**

Ring	Q	θ	φ
C7–C12	0.586	179.6	279.2
C13–C18	0.574	2.5	345.0
C19–C24	0.577	178.2	220.7
